# A case of prolonged corneal sulfur foreign bodies and review of literature

**DOI:** 10.1186/s12886-024-03571-x

**Published:** 2024-07-17

**Authors:** Kaijin Zheng, Xiaodong Li, Xuewei Qin, Hang Long, A-Ping Wu

**Affiliations:** 1 Ophthalmology Department of the First Affiliated Hospital of Guizhou, University of Traditional Chinese Medicine, Guiyang, China; 2 Department of Ophthalmology, Xi’an First Hospital, The First Affiliated Hospital of Northwest University, Xi’an, Shaanxi Province 710002 China

**Keywords:** Corneal foreign body, Sulphur mine, Inert metal foreign body

## Abstract

**Background:**

This case mainly describes a relatively rare case of an old mineral-like corneal foreign body that existed for up to 20 years, and did not significantly affect the visual quality of the patient.

**Case presentation:**

A 56-year-old male miner complained of right eye vision loss for 3 years, swollen and painful for 4 months. Admission examination: Best corrected visual acuity was no light perception in the right eye and 20/20 in the left eye. Anterior segment examination: A large number of spot-like grayish-brown mineral foreign bodies in the conjunctiva of the nasal conjunctiva, emulsified silicone oil floating in the anterior chamber, Corneal foreign bodies in the right eye were widely distributed in the upper cortex and the proelastic layer. There were fewer foreign bodies in the left cornea. Previous medical history, 20 years ago due to forging and burning sulphur mine explosion, resulting in a large number of ore foreign bodies in the conjunctiva of both eyes. As these corneal foreign bodies did not affect the visual quality of the patient, we adopted a conservative treatment plan, did not remove these foreign bodies, and only carried out symptomatic treatment for the patient’s secondary ocular hypertension. The patient was followed up normally in the outpatient department, and no cornea-related complications occurred up to now.

**Conclusions:**

First of all, it is necessary to understand the source and nature of the foreign body in patients with corneal and conjunctival foreign body injuries. In the second, for the old corneal metal foreign body, when the patient’s visual acuity is stable and there are no symptoms of corneal irritation and inflammatory reaction, it can be Conservative treatment or outpatient follow-up observation. In the end, corneal Optical coherence tomography imaging should not be ignored, which is very important for determining the depth of embedding and the location of the corneal foreign body.

## Background

The patient was admitted to the hospital for secondary glaucoma in the right eye, but this article focuses on the special manifestations of this case, in which a large number of metal and ore foreign bodies were present on the corneal conjunctiva of both eyes after the explosion of the sulfur mine, which lasted for nearly 20 years without secondary infections of the cornea and conjunctiva, and there have been no documented reports on the multiple corneo-conjunctival foreign body injuries after the explosion of the sulfur mine in the literature. Because of the long time span, the patient was unable to express the specific type of calcined sulfur ore at that time, and sulfur ore in China mainly contains inert metal elements such as “gold”, “silver”, “copper” and other inert metal elements [1], and secondly, the patient was unable to express the specific type of calcined sulfur ore at that time, According to the patient’s corneal conjunctiva foreign body retention time, it is presumed that the sulfur mine explosion at the time after the adhesion to the corneal conjunctiva of the foreign body may be mainly the inert metal and part of the gravel residue and other non-metallic substances. The inert metal and non-metallic foreign bodies in the superficial corneal layer, such as glass and sand, are chemically more stable and not easy to react with corneal tissues, so they can be retained in the corneal conjunctiva for a long time, and can be treated conservatively in the absence of irritating symptoms, secondary infections, and loss of visual function, which is in line with the clinical manifestations of this case. Patient received eye injury and intraocular foreign body due to an expolsion of calcined sulfur ore. Calcination is the process in which the ore of the metal is heated to a high temperature in the absence or limited supply of air or oxygen. Sulfide minerals oxidize rapidly when broken and exposed to air and, in operations where such minerals become dispersed as dusts, sparks or heat flash from blasting can initiate an explosion. The consequences can range from mine pollution by poisonous clouds of sulfurous gases (Sulfur dioxide and Hydrogen sulfide). These can cause eyes and throat irritation, at times leads to difficulty in breathing and even to loss of life. Previous research has shown that the effect of sulfur (endogenous and exogenous) on human tissue and eyes (cornea, conjunctiva, iris and trabecular meshwork), Kravchik MV et al [2]found that In patients with primary open-angle glaucoma, an increase in the intraocular pressure level causes the amount of sulfur associated with pigment granules and the proportion of organic phosphorus to increase in the trabecular meshwork, which should be taken into account in the further search for drug therapy that would potentially affect pathologically altered tissue. The authors postulate that higher sulfur contact in trabecular meshwork was responsible for glaucoma. The latest literature [3] reports a goldsmith with a large number of gold particles embedded in the corneal epithelial and stromal layers of both eyes, most of which had disappeared after receiving 1% prednisone acetate and 0.5% carboxymethylcellulose artificial tears, and with a significant improvement in visual acuity.

## Case presentation

**Fig. 1:**
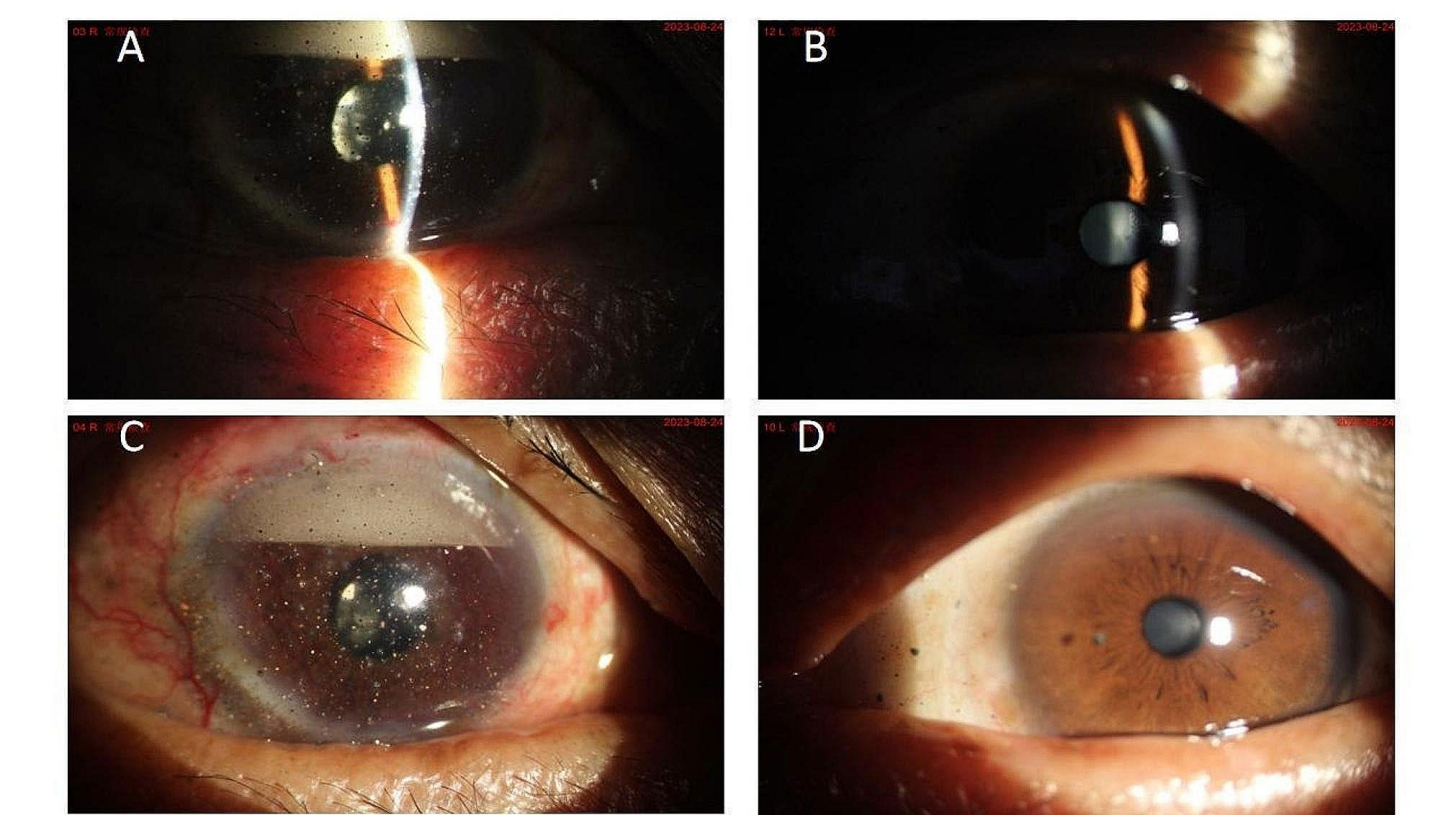
Anterior segment photography of both eyes with multiple old corneal foreign bodies after a sulfur mine explosion 20 years ago; **A** and **C** show slit-lamp anterior segment images of the right eye, with a large number of golden-yellow, silvery-white, and gray-black granular foreign bodies densely distributed in the corneo-conjunctiva, emulsified silicone oil visible in the anterior chamber of the right eye, and a network of neovascularization on the surface of the iris, with the IOL in place; **B** and **D** show slit-lamp anterior segment images of the left eye, with yellow and gray-black foreign bodies outside the pupil area of the cornea and on the conjunctiva. granular foreign body, anterior chamber (-), and slightly cloudy crystalline lens

**Fig. 2:**
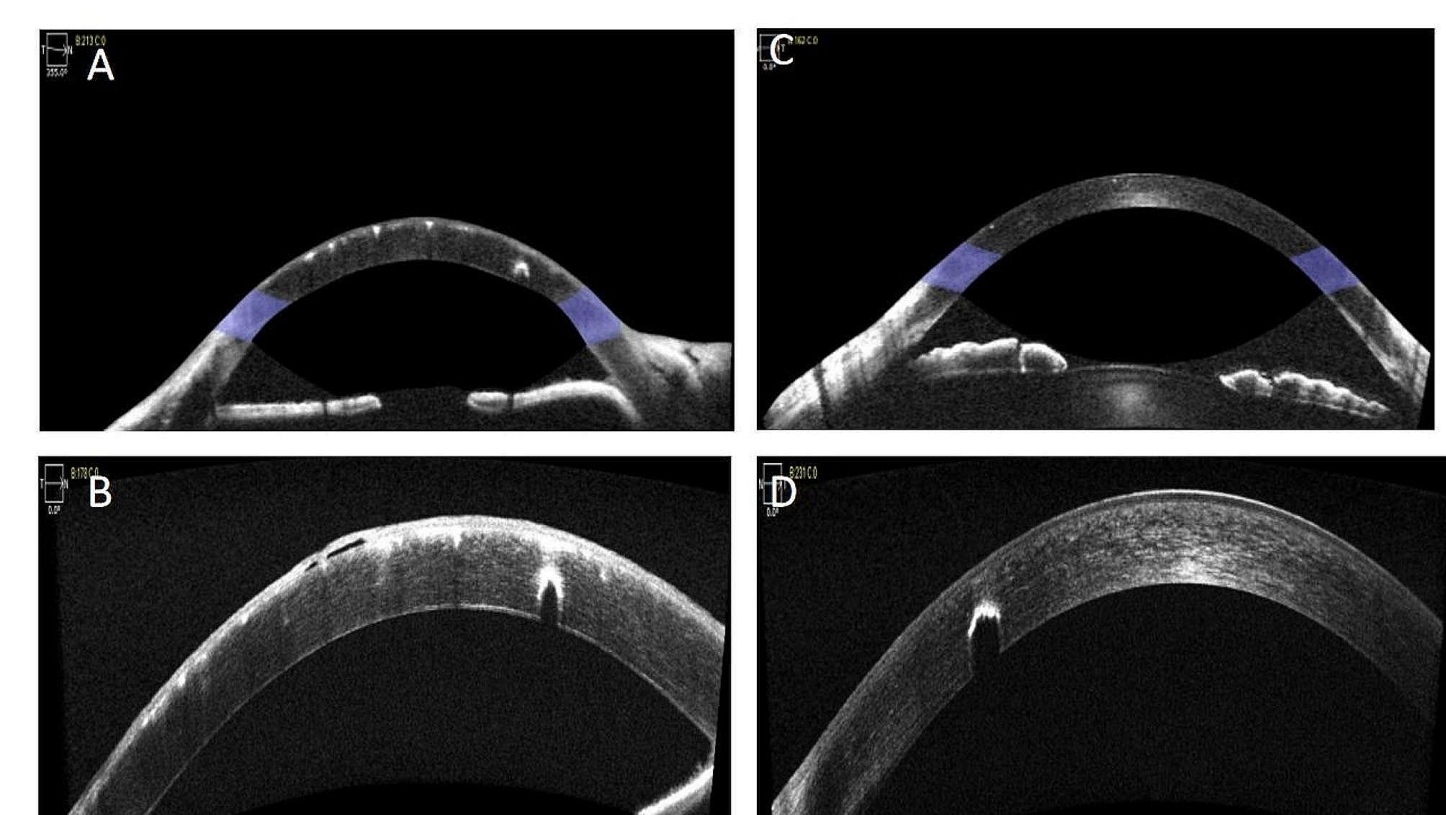
OCT images of the anterior segments of both eyes with multiple old corneal foreign bodies after a sulfur mine explosion; **A**: shows the overall OCT image of the right cornea, with a large number of punctate and clustered hyperreflective signals from the corneal epithelium, anterior elastic lamina, and stroma, and the depth of the anterior chamber is normal, and the angle of the anterior chamber is open, **B**: shows a partial OCT image of the right cornea, with a large number of punctate hyperreflective signals from the corneal epithelium, anterior elastic lamina, and stroma and a small low-reflective signal lumen between the cornea’s anterior elastic lamina as well as a small low-reflective signal lumen between the anterior elastic lamina and the stroma (consider a localized detachment of the corneal anterior elastic lamina due to a prolonged period of high intraocular pressure).**C**: shows the overall OCT image of the left anterior segment, with scattered high reflection points visible in the shallow cornea, open atrial corners, and normal anterior chamber depth, **D**: shows a local OCT image of the cornea of the left eye. A curved band of highly reflective signals can be seen in the corneal stromal layer with low signal occlusion and normal corneal thickness

A 56 years old male patient was admitted to hospital due to “decreased vision in the right eye for 3 + years, aggravated distension and eye pain for 4 + months”. The patient underwent “vitrectomy of right eye combined with silicone oil filling” and “cataract removal by ultrasonography combined with IOL implantation in the right eye” for retinal detachment 3 years ago in a foreign hospital. 4 months ago, the patient lost vision in the right eye, accompanied by ocular swelling and pain in the right side of the head. The patient was admitted to the hospital with the following symptoms: loss of vision in the right eye, accompanied by ocular swelling and pain and right side of the head. Upon admission, he had no light perception in the right eye and the best corrected visual acuity of 1.0 in the left eye; intraocular pressure of 43 mmHg in the right eye and 16 mmHg in the left eye; a large number of dots and flakes of grayish-brown foreign bodies were seen in the corneal conjunctiva of both eyes; the conjunctiva of the right eye was congested and a large amount of emulsified silicone oil could be seen in the anterior chamber floating above it; there were large amounts of neovascularization networks on the surface of the iris, pupils were round, light reflex was absent, position of the IOL was centered, vitreous was cloudy, and there was no funduscopic vision, the left anterior chamber of the eye was shallowed, the crystalline lens was slightly cloudy, and the fundus was normal. Further questioning of the patient’s medical history, he complained that 20 + years ago, a foreign body entered his eyes due to an explosion of calcined sulfur ore, and he was discharged from the local hospital after undergoing a “bilateral corneal membrane foreign body removal surgery”, and his binocular vision was unaffected, with occasional astringency and discomfort, and he was perfected with auxiliary examinations: anterior ocular segment radiography showed that Anterior segment photography showed a large number of golden yellow, silver-white and gray-black granular foreign bodies on the corneal conjunctiva of both eyes, emulsified silicone oil in the anterior chamber of the right eye, neovascularization on the iris surface, and IOL in place, while the lens of the left eye was cloudy (shown in Fig. 1A-D); Optical Coherence Tomography (cornea) showed that the epithelium, anterior elastic lamina, and the stroma of the cornea of both eyes showed punctate and granular hyperreflective signals (shown in Fig. 2A-D); Fundus photography showed that the right eye could not be peeped into, the left eye had a clear optic disc, a temporal arcuate spot around the disc, and a flat retina, Optical Coherence Tomography (macula) showed that the interlayer structure of the retina in the right eye was not affectionate, and the left eye had a macular anterior membrane, Ocular ultrasound showed diffuse punctate moderate to high echogenic signal in the vitreous cavity of the right eye, and few punctate hypoechoic signals in the vitreous cavity of the left eye. The preliminary diagnosis was: Secondary glaucoma in the right eye (neovascular glaucoma), Silicone oil eye in the right eye, Foreign body in the corneal conjunctiva of both eyes, IOL eye in the right eye, Macular anterior membrane in the left eye. Diagnosis and treatment: The patient refused to undergo silicone oil removal surgery for the time being, and asked for relief of the right eye swelling and pain and lowering intraocular pressure. After completing the preoperative preparations, the patient underwent peripheral iridectomy with anterior chamber flushing of the right eye, and after the surgery, the patient’s right eye distension was relieved significantly, and the intraocular pressure of the right eye was measured to be 15 mm Hg. Considering that the patient had no light perception in the right eye and visual acuity in the left eye was 1.0, the foreign body in the left eye was outside the pupil area, and there was no rust ring embroidery stains and necrotic foci of infection around the foreign body in both eyes, therefore, no intervention was made to remove the foreign body from both eyes for the time being, and the patient was temporarily given gatifloxacin ophthalmic gel and sodium hyaluronate eye drops and was given a follow-up examination after half a month.

## Discussion and conclusions

The cornea is the most innervated tissue in the human body, rich in sensory nerve tissue, the corneal epithelial cells are densely distributed between the ocular branch of the Vth pair of cerebral nerves, and the sensation is sensitive. Superficial corneal foreign body is easy to stimulate the sensory nerves of corneal epithelial layer, corneal irritation symptoms such as foreign body sensation, pain and photophobia, vision loss, large amount of tears, blepharospasm, and conjunctival congestion, etc. These patients tend to seek medical treatment in time, and the prognosis is good. The corneal stroma has no sensory nerve distribution, so when the foreign body reaches the stroma, it is not easy to be felt by the patient, and the patient will only seek medical treatment when secondary infection or vision loss occurs, resulting in a poor prognosis due to the lack of timely treatment [4]. In particular, corneal metal foreign body injury as a common disease in ophthalmic emergency, grasp the timing of metal foreign body removal is very important, the best time window for treatment is within 30 min after injury, especially iron foreign body into the cornea, the role of CO_2_ formation of ferrous iron bicarbonate, the hardness decreases easy to fracture and dispersal, more than 6 h after oxidation reaction, the formation of rust ring, the difficulty of rejection increased significantly, and rust ring easy to lead to the infiltration of corneal tissue, softening of the cornea. Corneal tissue infiltration, softening, necrosis and other changes, further aggravating the damage to normal corneal tissue [5, 6]. In this case, no softened or necrotic corneal tissue was found around the metallic corneal foreign body, which excludes the possibility that the corneal foreign body was a ferrous metallic foreign body.

The current study [7] classifies corneal metal foreign body injuries into three main grades according to the depth of foreign body adhesion and clinical manifestations: I degree: the depth of adhesion of the metal foreign body does not exceed the anterior elastic lamina, there are no rust stains at the base, and there is no rust ring; II degree: the depth of adhesion of the metal foreign body is up to and does not exceed the stromal layer, there is no obvious rust ring around the foreign body and there are a few rust stains in the stromal layer; III degree: the depth of adhesion of the metal foreign body is more than the stromal layer, but there is not a complete perforation or a rust ring formed around the foreign body. Corneal foreign body area [8]: taking the midpoint of the iris as the radius and drawing a circle around the center of the pupil, the cornea was divided into 3 zones, Zone A: the corneal area corresponding to the line between the midpoints of the iris. Zone B: between Zone A and Zone C. Zone C: the area corresponding to the pupil. According to the above classification and the results of anterior segment photography and corneal OCT, most of the foreign bodies in both eyes were adhered to the corneal epithelium and anterior elastic lamina, which was classified as degree I damage, and some foreign bodies were embedded in the corneal stroma, which was classified as degree II damage.

The prognosis of different corneal metal foreign body damage degree is also different, I degree damage foreign body removal cornea can be repaired in a short period of time, almost does not affect vision, this is because the depth of damage is not more than the anterior elastic lamina, and at the same time the repair and regeneration of corneal epithelial cells is strong. II and III degrees of damage to take out the foreign body after the removal of the residual corneal opacity or corneal opacities, easy to cause corneal astigmatism affecting vision, this is related to the destruction of the stroma layer of corneal tissue This is related to the tissue proliferation after the corneal stromal layer is damaged. And when the active metal will oxidize and precipitate rust stains into the corneal tissue to form a rust ring, which can not be clearly distinguished from the normal tissue, so it is not easy to remove the foreign body, and the scope of damage is large, and the cornea will be repaired for a long time, and it is easy to be infected, and the scar will be obvious after the healing process. Corneal foreign body removal surgery with recombinant human epidermal growth factor eye drops [9], calf blood deproteinized extract ophthalmic gel and corneal bandage lens [10] to promote corneal epithelial repair, glucocorticoid drops such as tobramycin and dexamethasone eye drops for the treatment of postoperative corneal edema [11], as well as antibiotics, such as levofloxacin eye drops to prevent postoperative infections [12], for corneal metal foreign body, can be combined with the application of 0.37% of the disodium edetate eye drops make the Separation of rust in corneal tissue [13], which is beneficial to the removal of rust rings and rust spots.

The main lessons learned through this case are summarized as follows: (1) First of all, it is necessary to understand the source and nature of the foreign body in patients with corneal and conjunctival foreign body injuries. This case is special, the time span is too long, the patient’s expression of the type of sulfur ore is unclear, combined with the clinical manifestations and imaging results, the main foreign body components are considered to be inert metals such as gold, silver and copper, and non-metallic substances such as sand and gravel, the prolonged retention of sulfur mineral foreign bodies do not cause necrosis of corneal tissue. so that it has not caused corneal infection and necrosis complications such as iron deposits in the past 20 years; (2)In the second place, for the old corneal metal foreign body, when the patient’s visual acuity is stable and there are no symptoms of corneal irritation and inflammatory reaction, it can be Conservative treatment or outpatient follow-up observation, especially the foreign body rejection located in the stromal layer may be secondary to corneal scarring or infection, resulting in corneal astigmatism and visual dysfunction; (3)In the end, corneal OCT imaging should not be ignored, which is very important for determining the depth of embedding and the location of the corneal foreign body.

## Data Availability

All data and images can be obtained by contacting the corresponding author.
